# Supplementary figures and images for: Risk factors for in-hospital mortality in laboratory-confirmed COVID-19 patients in the Netherlands: A competing risk survival analysis
Source: PLoS One. 2021 Mar 26;16(3):e0249231. doi: 10.1371/journal.pone.0249231 (PMC7997038; doi:10.1371/journal.pone.0249231)

**S1 Fig. Flowchart of inclusion**

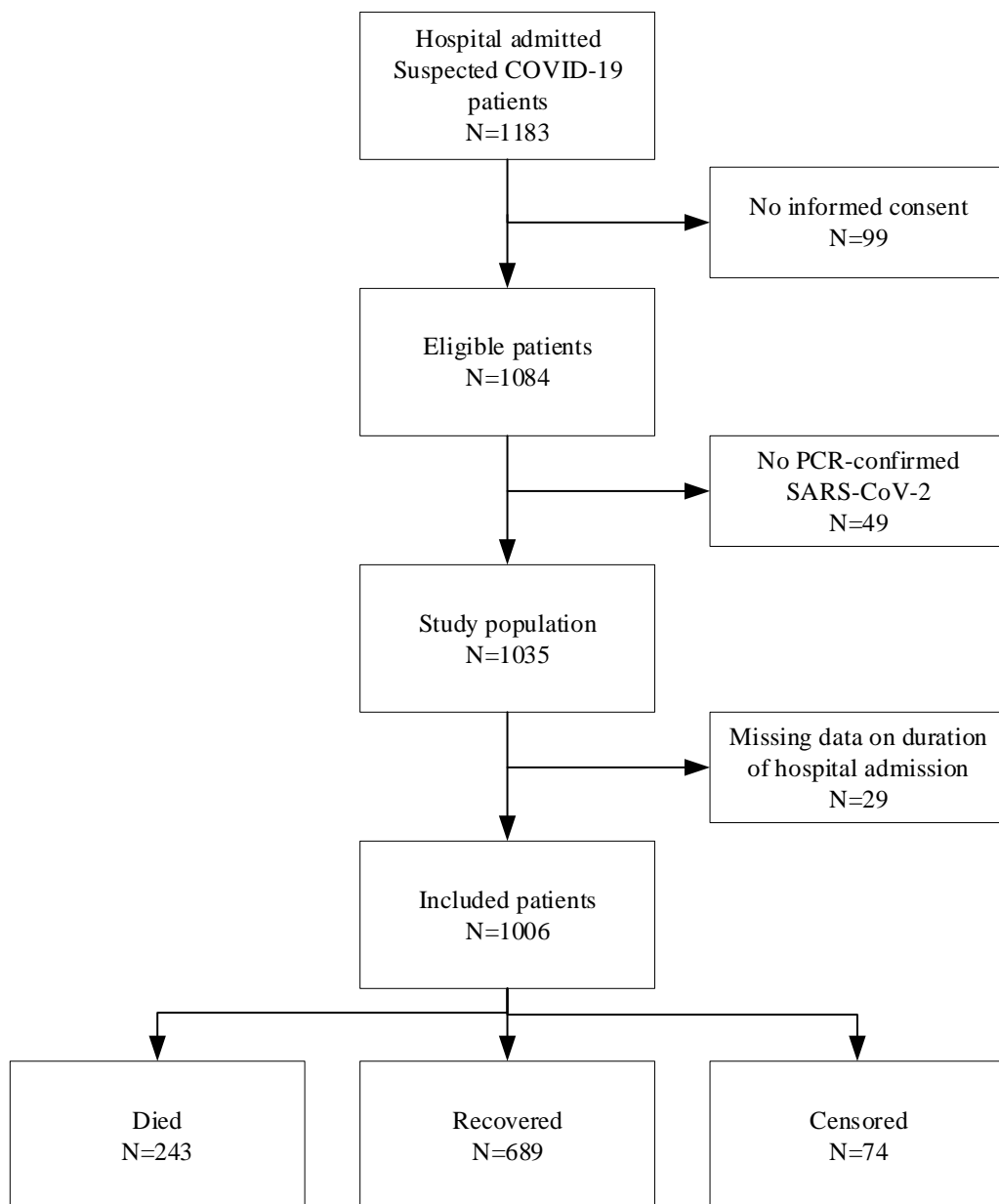

Supplement: S1 Fig — (PDF) [file pone.0249231.s002.pdf]
