# Supplementary material for: Risk factors for in-hospital mortality in laboratory-confirmed COVID-19 patients in the Netherlands: A competing risk survival analysis
Source: PLoS One. 2021 Mar 26;16(3):e0249231. doi: 10.1371/journal.pone.0249231 (PMC7997038; doi:10.1371/journal.pone.0249231)
Supplement: S2 Fig — A. Plots of the Schoenfeld residuals for the multivariable CSH model for death. B. Plots of the Schoenfeld residuals for the multivariable CSH model for recovery. (PDF) [file pone.0249231.s003.pdf]

S2 Fig. Schoenfeld residuals plots

A. Plots of the Schoenfeld residuals for the multivariable CSH model for death.

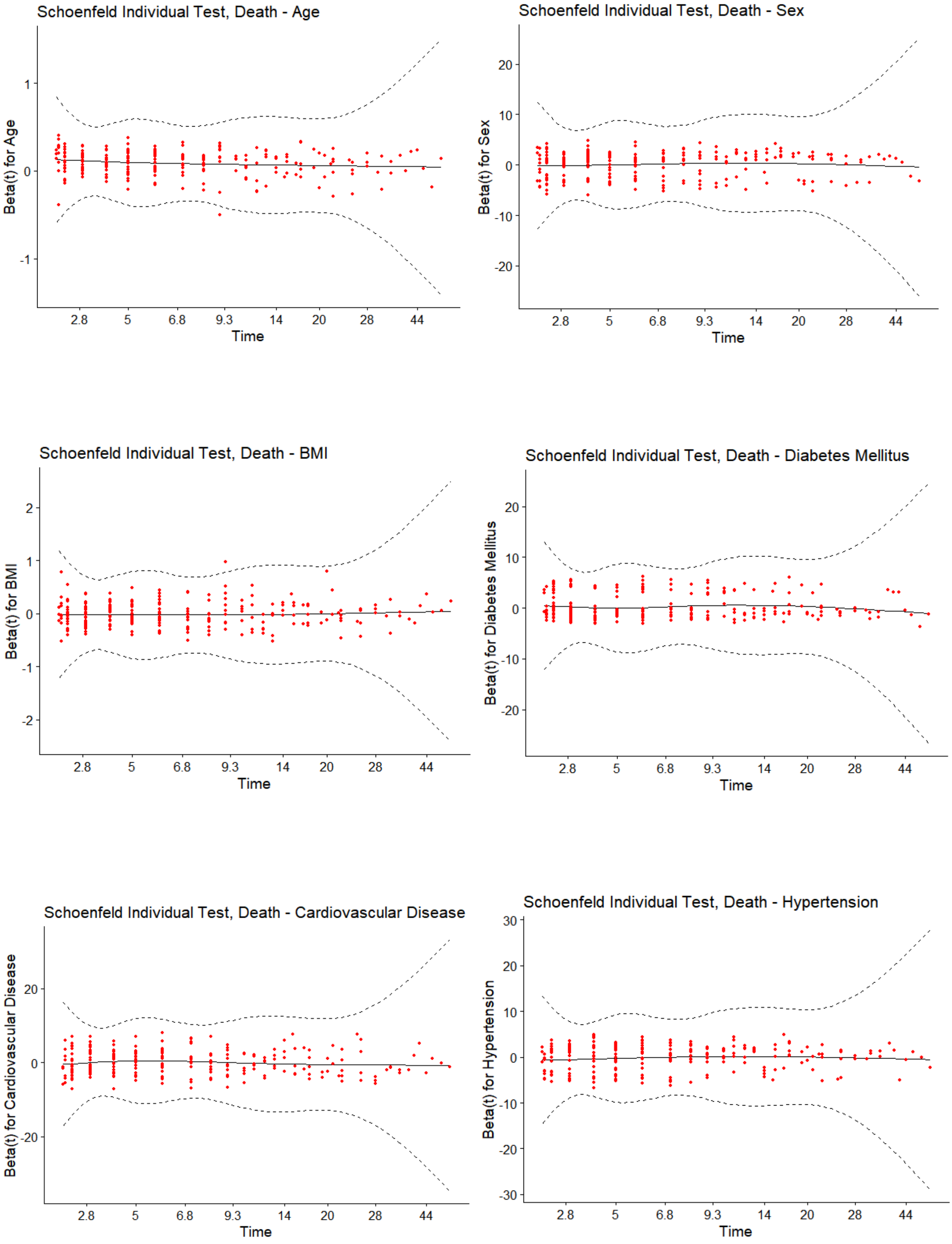

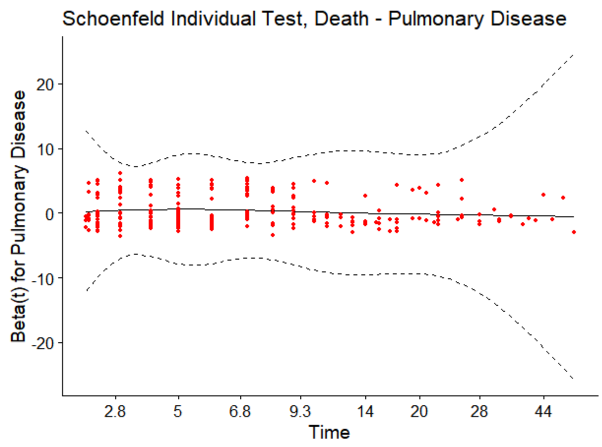
Schoenfeld Individual Test, Death - Pulmonary Disease
Beta(t) for Pulmonary Disease
20
10
0
-10
-20
2.8
5
6.8
9.3
14
20
28
44
Time

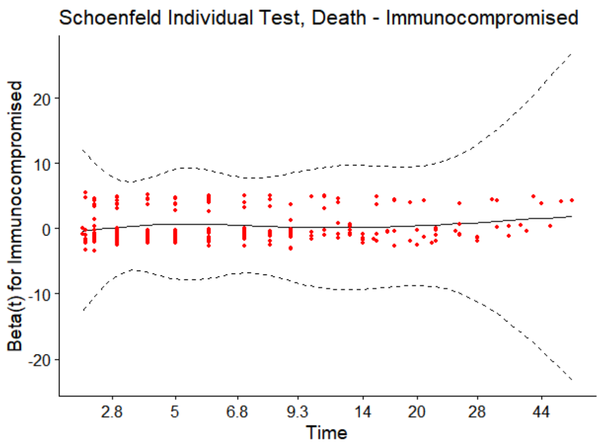
Schoenfeld Individual Test, Death - Immunocompromised
Beta(t) for Immunocompromised
20
10
0
-10
-20
2.8
5
6.8
9.3
14
20
28
44
Time

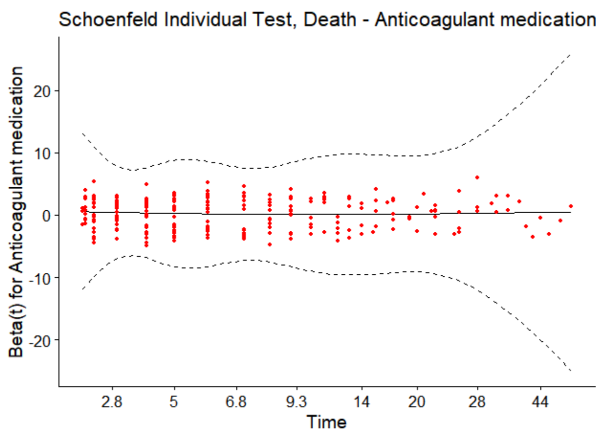
Schoenfeld Individual Test, Death - Anticoagulant medication
Beta(t) for Anticoagulant medication
20
10
0
-10
-20
2.8
5
6.8
9.3
14
20
28
44
Time

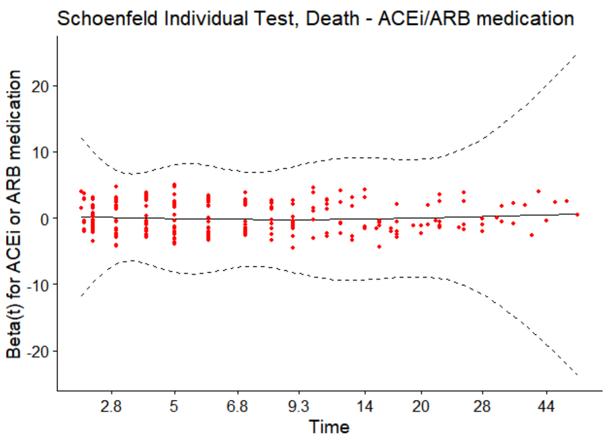
Schoenfeld Individual Test, Death - ACEi/ARB medication
Beta(t) for ACEi or ARB medication
20
10
0
-10
-20
2.8
5
6.8
9.3
14
20
28
44
Time

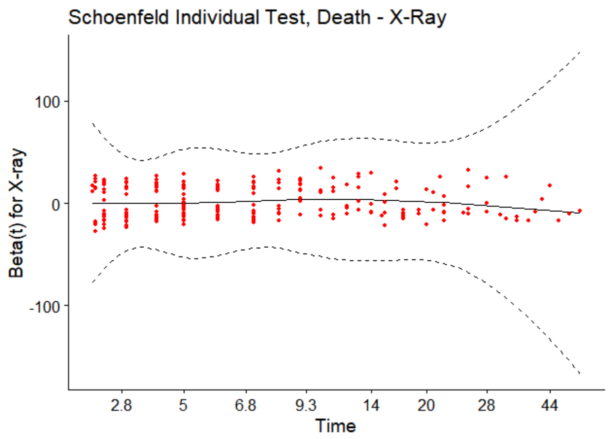
Schoenfeld Individual Test, Death - X-Ray
Beta(t) for X-ray
100
0
-100
2.8
5
6.8
9.3
14
20
28
44
Time

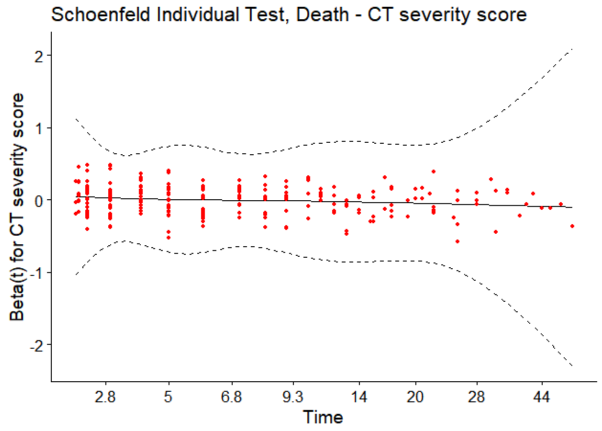
Schoenfeld Individual Test, Death - CT severity score
Beta(t) for CT severity score
2
1
0
-1
-2
2.8
5
6.8
9.3
14
20
28
44
Time

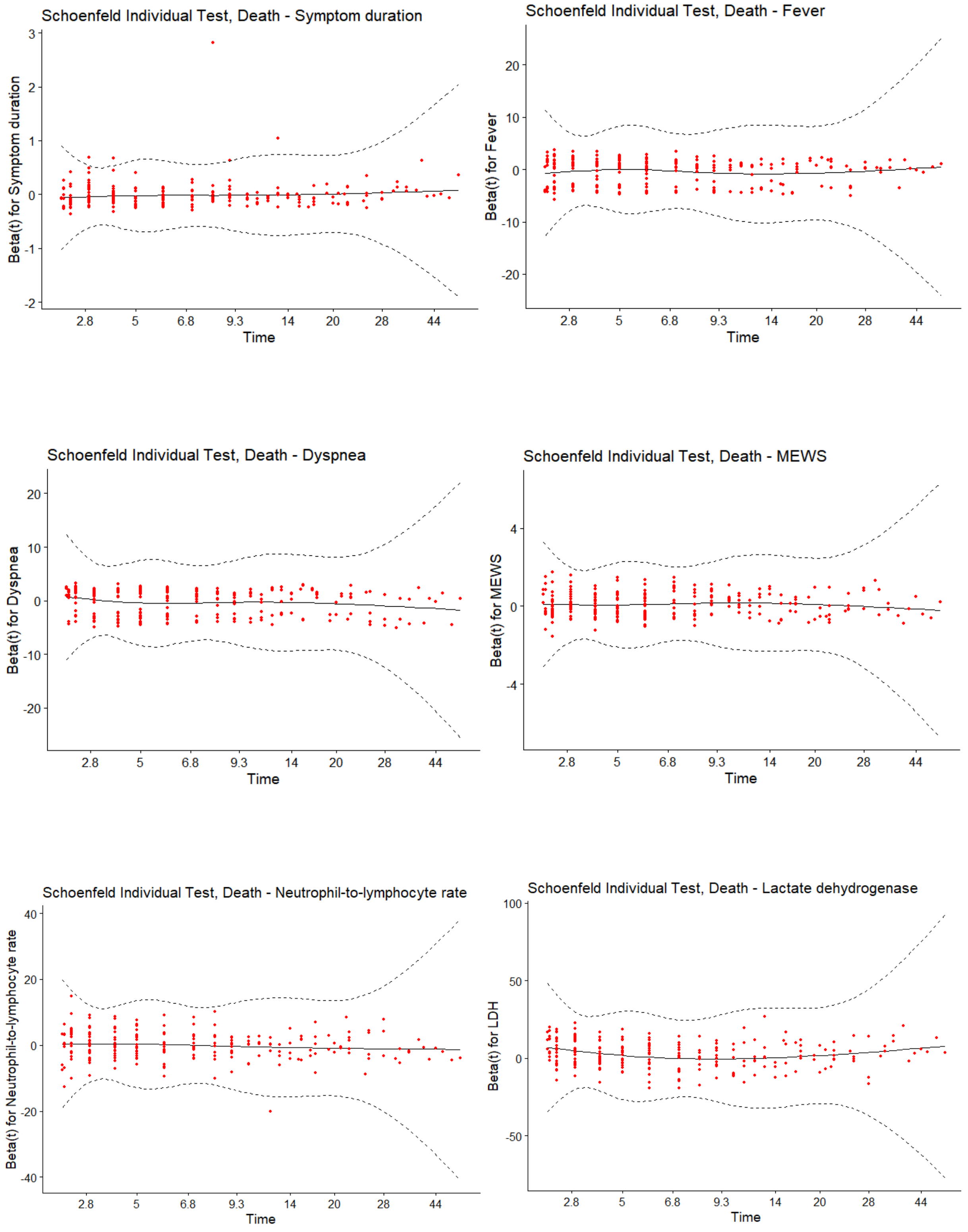
Schoenfeld Individual Test, Death - Symptom duration
Beta(t) for Symptom duration
Time
Schoenfeld Individual Test, Death - Fever
Beta(t) for Fever
Time
Schoenfeld Individual Test, Death - Dyspnea
Beta(t) for Dyspnea
Time
Schoenfeld Individual Test, Death - MEWS
Beta(t) for MEWS
Time
Schoenfeld Individual Test, Death - Neutrophil-to-lymphocyte rate
Beta(t) for Neutrophil-to-lymphocyte rate
Time
Schoenfeld Individual Test, Death - Lactate dehydrogenase
Beta(t) for LDH
Time
2.8
5
6.8
9.3
14
20
28
44

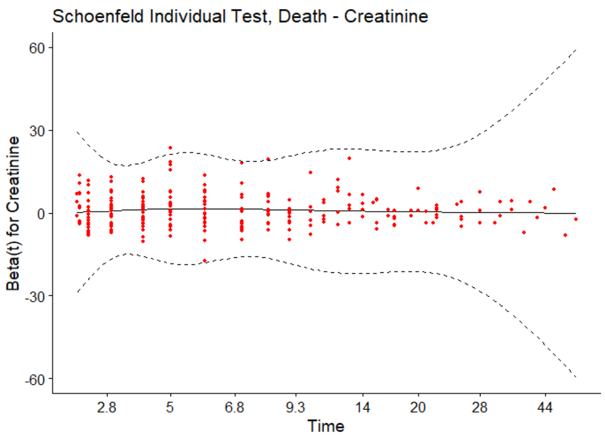
Schoenfeld Individual Test, Death - Creatinine
Beta(t) for Creatinine
60
30
0
-30
-60
2.8
5
6.8
9.3
14
20
28
44
Time

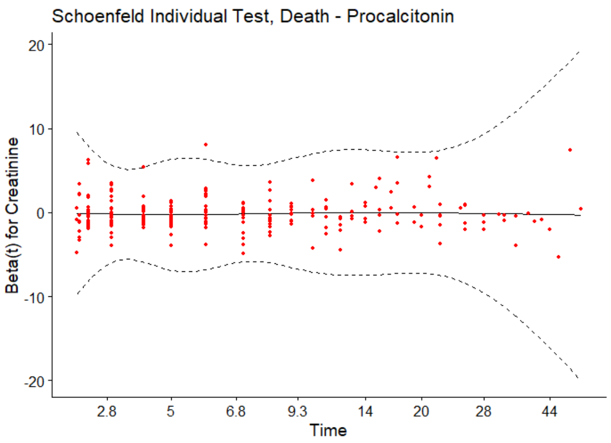
Schoenfeld Individual Test, Death - Procalcitonin
Beta(t) for Creatinine
20
10
0
-10
-20
2.8
5
6.8
9.3
14
20
28
44
Time

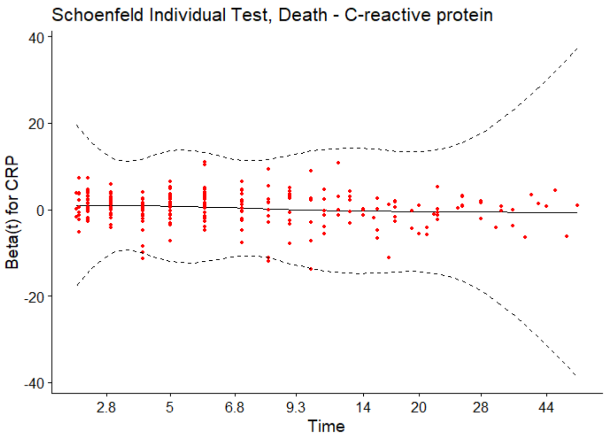
Schoenfeld Individual Test, Death - C-reactive protein
Beta(t) for CRP
40
20
0
-20
-40
2.8
5
6.8
9.3
14
20
28
44
Time

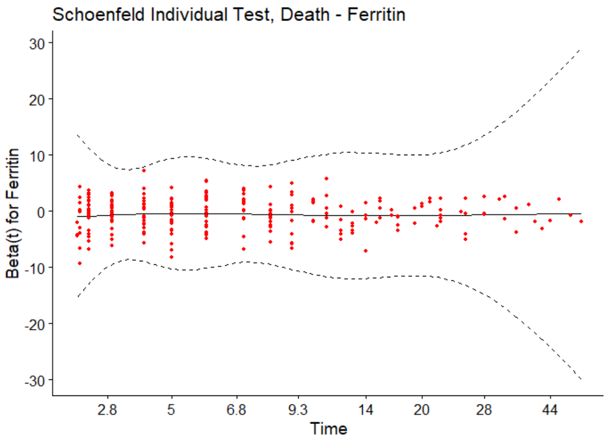
Schoenfeld Individual Test, Death - Ferritin
Beta(t) for Ferritin
30
20
10
0
-10
-20
-30
2.8
5
6.8
9.3
14
20
28
44
Time

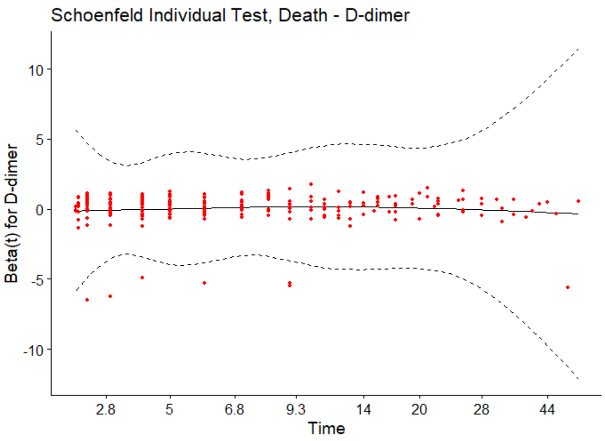
Schoenfeld Individual Test, Death - D-dimer
Beta(t) for D-dimer
10
5
0
-5
-10
2.8
5
6.8
9.3
14
20
28
44
Time

B. Plots of the Schoenfeld residuals for the multivariable CSH model for recovery.

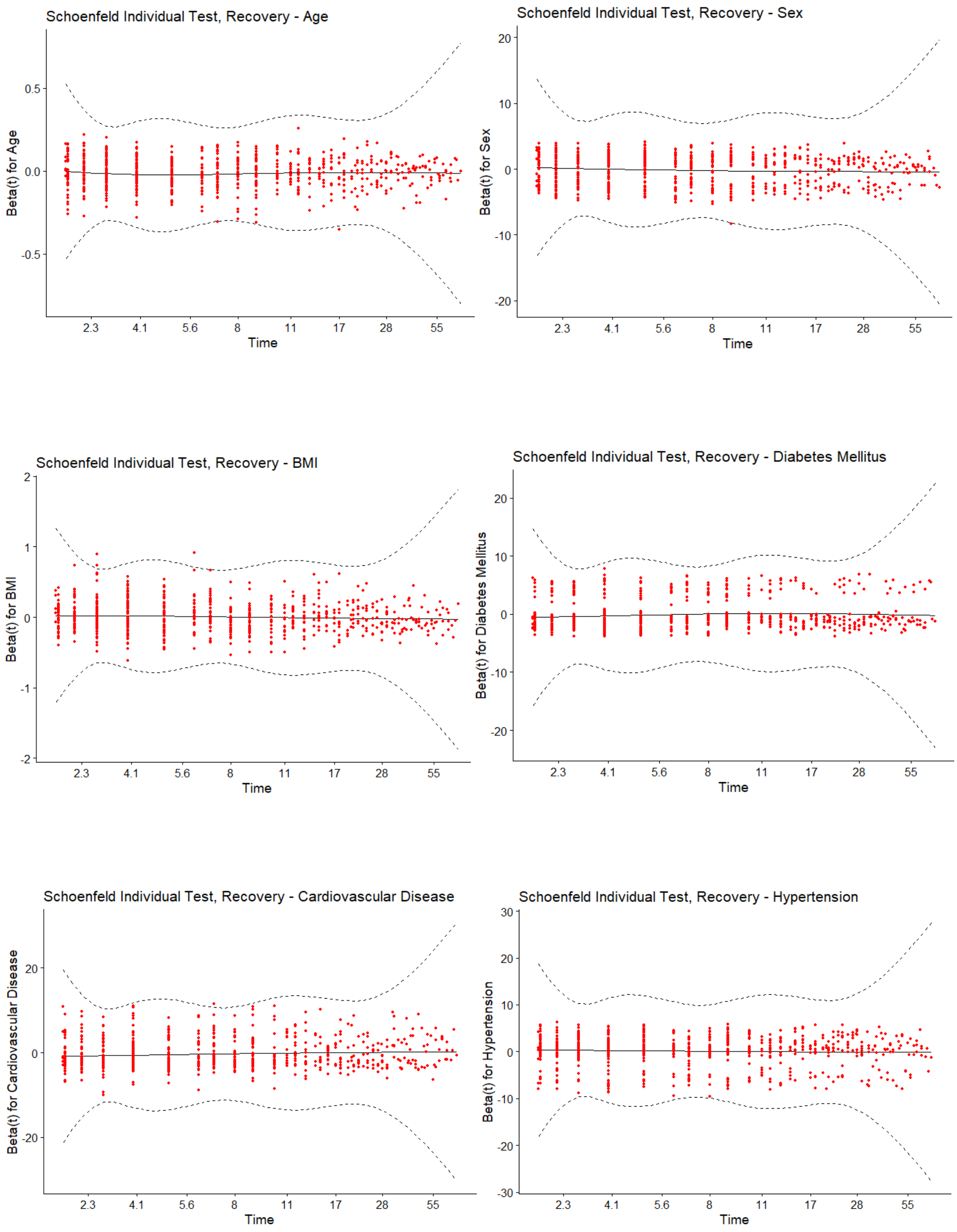

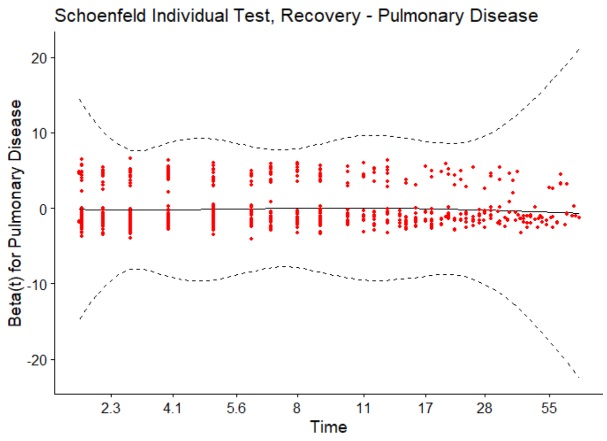
Schoenfeld Individual Test, Recovery - Pulmonary Disease
Beta(t) for Pulmonary Disease
20
10
0
-10
-20
2.3
4.1
5.6
8
11
17
28
55
Time

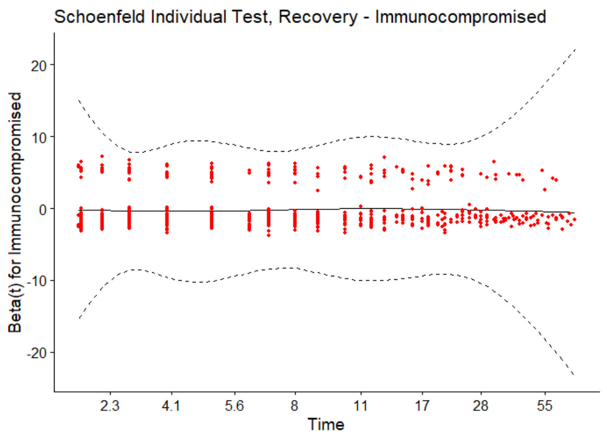
Schoenfeld Individual Test, Recovery - Immunocompromised
Beta(t) for Immunocompromised
20
10
0
-10
-20
2.3
4.1
5.6
8
11
17
28
55
Time

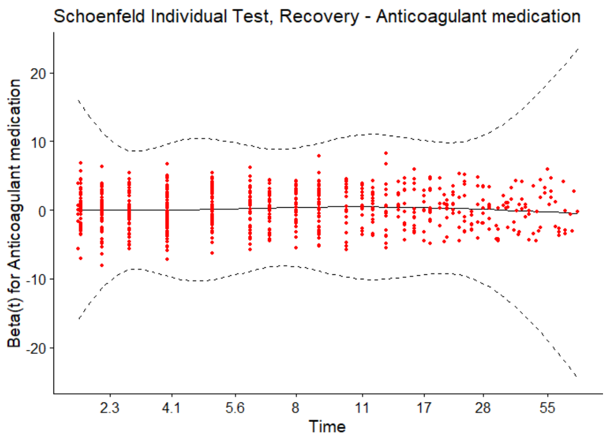
Schoenfeld Individual Test, Recovery - Anticoagulant medication
Beta(t) for Anticoagulant medication
20
10
0
-10
-20
2.3
4.1
5.6
8
11
17
28
55
Time

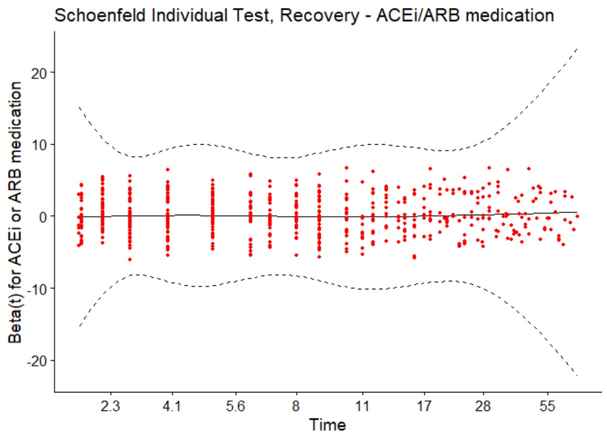
Schoenfeld Individual Test, Recovery - ACEi/ARB medication
Beta(t) for ACEi or ARB medication
20
10
0
-10
-20
2.3
4.1
5.6
8
11
17
28
55
Time

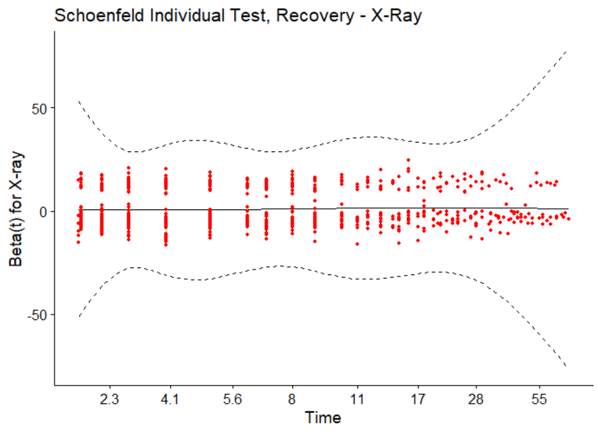
Schoenfeld Individual Test, Recovery - X-Ray
Beta(t) for X-ray
50
0
-50
2.3
4.1
5.6
8
11
17
28
55
Time

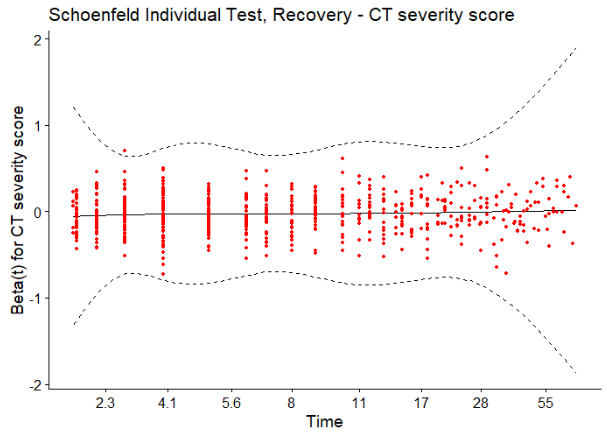
Schoenfeld Individual Test, Recovery - CT severity score
Beta(t) for CT severity score
2
1
0
-1
-2
2.3
4.1
5.6
8
11
17
28
55
Time

Schoenfeld Individual Test, Recovery - Symptom duration

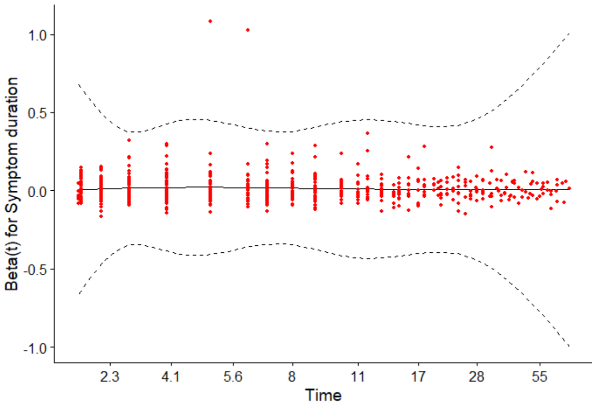

Schoenfeld Individual Test, Recovery - Fever

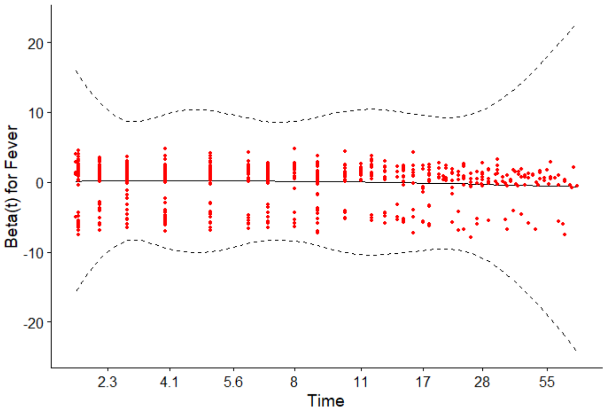

Schoenfeld Individual Test, Recovery - Dyspnea

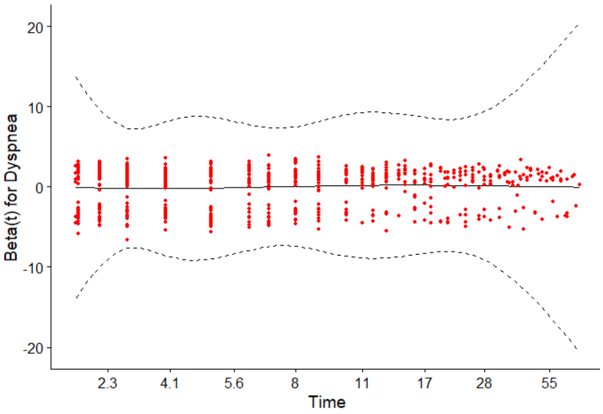

Schoenfeld Individual Test, Recovery - MEWS

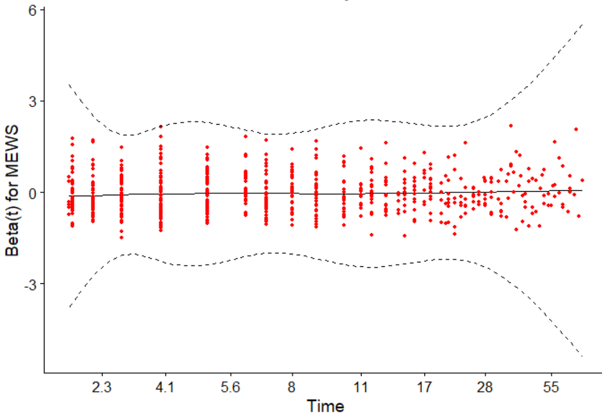

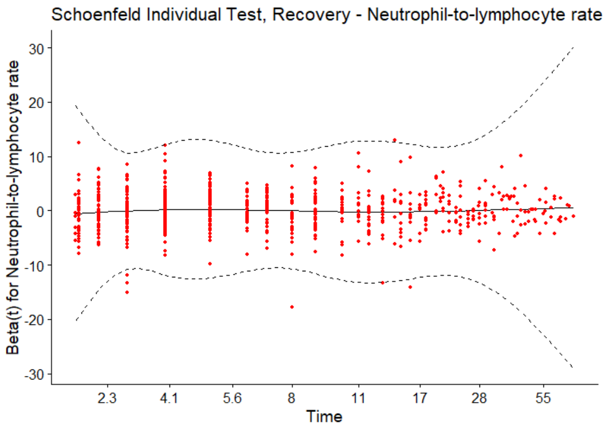

Schoenfeld Individual Test, Recovery - Neutrophil-to-lymphocyte rate
Beta(t) for Neutrophil-to-lymphocyte rate
30
20
10
0
-10
-20
-30
2.3
4.1
5.6
8
11
17
28
55
Time

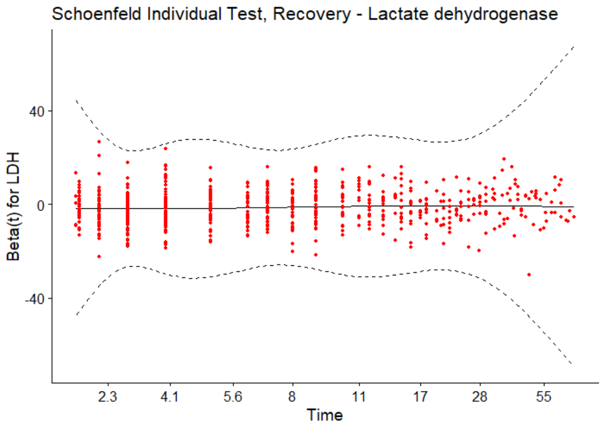

Schoenfeld Individual Test, Recovery - Lactate dehydrogenase
Beta(t) for LDH
40
0
-40
2.3
4.1
5.6
8
11
17
28
55
Time

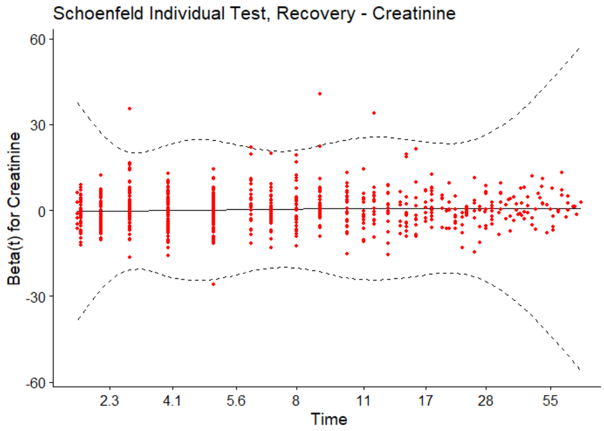
Schoenfeld Individual Test, Recovery - Creatinine
Beta(t) for Creatinine
60
30
0
-30
-60
2.3
4.1
5.6
8
11
17
28
55
Time

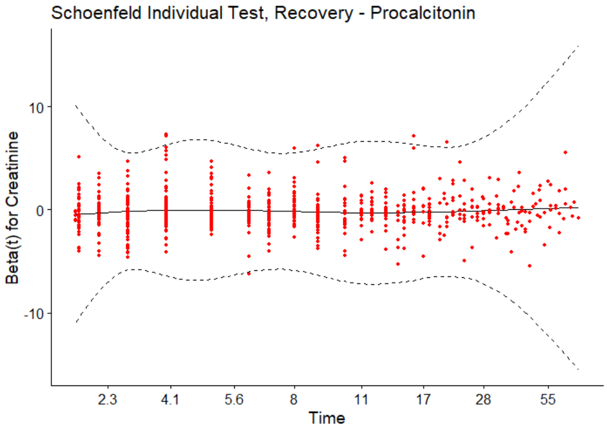
Schoenfeld Individual Test, Recovery - Procalcitonin
Beta(t) for Creatinine
10
0
-10
2.3
4.1
5.6
8
11
17
28
55
Time

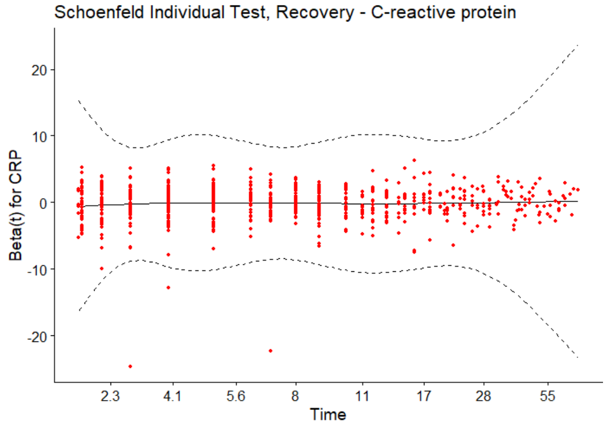
Schoenfeld Individual Test, Recovery - C-reactive protein
Beta(t) for CRP
20
10
0
-10
-20
2.3
4.1
5.6
8
11
17
28
55
Time

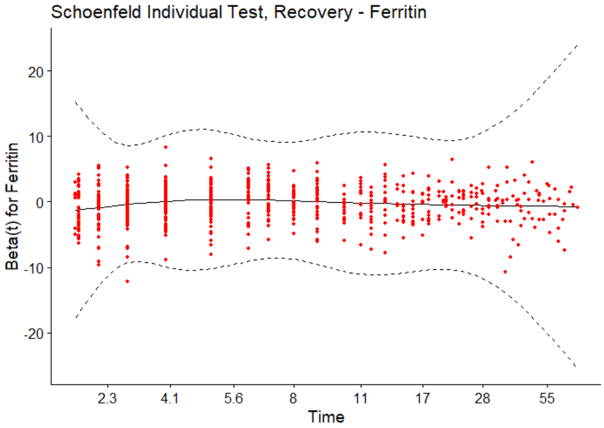
Schoenfeld Individual Test, Recovery - Ferritin
Beta(t) for Ferritin
20
10
0
-10
-20
2.3
4.1
5.6
8
11
17
28
55
Time

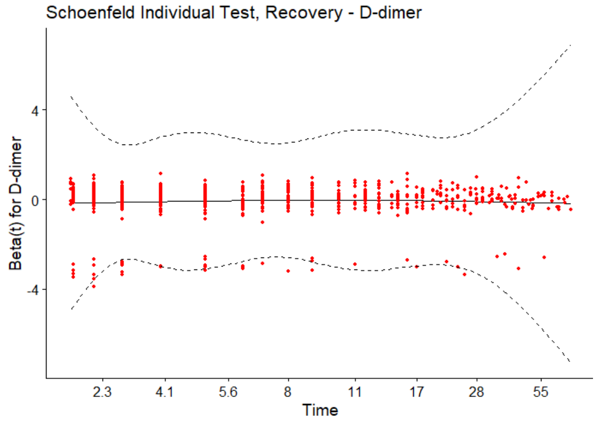
Schoenfeld Individual Test, Recovery - D-dimer
Beta(t) for D-dimer
4
0
-4
2.3
4.1
5.6
8
11
17
28
55
Time
